# DcR3-associated risk score: correlating better prognosis and enhanced predictive power in colorectal cancer

**DOI:** 10.1007/s12672-024-01082-1

**Published:** 2024-06-19

**Authors:** Ying Duan, Hangrong Fang, Juanhong Wang, Banlai Ruan, Juan Yang, Jie Liu, Siqi Gou, Yijie Li, Zhengyi Cheng

**Affiliations:** 1 Department of Pathology, Xi’an No. 3 Hospital, The Affiliated Hospital of Northwest University, Xi’an, 710018 Shaanxi People’s Republic of China; 2 CAS Key Laboratory of Quantitative Engineering Biology, Shenzhen Institute of Advanced Technology, Shenzhen Institute of Synthetic Biology, Chinese Academy of Sciences, Shenzhen, China; 3 Medical Research Center, Xi’an Key Laboratory of Cardiovascular and Cerebrovascular Diseases, Xi’an No. 3 Hospital, The Affiliated Hospital of Northwest University, Xi’an, Shaanxi China

**Keywords:** DcR3, Riskscore model, IL-17 signaling pathway, Colorectal cancer

## Abstract

Decoy receptor 3 (DcR3), a novel soluble protein belonging to the tumor necrosis factor receptor (TNFR) family, has been previously associated with tumorigenesis in various cancers. However, in our study, we unexpectedly found that DcR3 may promote patient survival time in colorectal cancer (CRC). Through an analysis of The Cancer Genome Atlas (TCGA) and Gene Expression Omnibus (GEO) datasets, we discovered that high levels of DcR3 are associated with improved overall survival (OS) and disease-free survival (DFS) in CRC patients. Further investigation revealed that DcR3 is correlated with favorable clinical features in Metastasis 0 (M0) and stage I/II CRC patients, suggesting it may act as a suppressive factor in CRC. Gene Set Enrichment Analysis (GSEA) demonstrated that the high DcR3 group is enriched in the IL-17 signaling pathway and other immune-related pathways, and Single Sample Gene Set Enrichment Analysis (ssGSEA) revealed a higher abundance of Tumor Infiltrating Lymphocytes (TIL) in the DcR3 high group. To better understand the function of DcR3, we constructed a DcR3-associated riskscore (DARS) model using machine learning, comprising three genes (DPP7, KDM3A, and TMEM86B). The DARS model indicated that high riskscore patients have an unfavorable prognosis, and it is associated with advanced stages (III/IV), T3/4 tumors, and N1/2 lymph node involvement. Additionally, high riskscore group exhibited more frequent gene mutations, such as TTN, MUC16, and SYNE1, with SYNE1 mutation being related to poor prognosis. Intriguingly, DcR3 showed higher expression in the low riskscore group. These results suggest that DcR3 could serve as a potential prognostic biomarker in CRC and may play a crucial role in favorably modulating the immune response in this malignancy.

**Supplementary Information:**

The online version contains supplementary material available at 10.1007/s12672-024-01082-1.

## Introduction

Colorectal cancer (CRC) represents a significant global health challenge, ranking among the most prevalent and lethal cancers worldwide. Its alarming incidence and mortality rates have prompted substantial research efforts to improve patient outcomes. Each year, more than 1.85 billion patients succumb to CRC, and over 850 thousand people receive new diagnoses [1]. Despite considerable investment in CRC treatment, many patients still face adverse outcomes due to inappropriate care or delayed interventions. The 5-year survival rate for CRC stands at approximately 64%, plunging to a mere 12% for metastatic CRC [2]. Consequently, there is an urgent need to develop more effective and efficient treatment approaches to enhance CRC patients' survival. Surgical resection remains the primary curative method for tumor removal, yet metastasis to distant tissues remains a major challenge [3, 4]. Once metastasis occurs, therapeutic options such as chemotherapy, radiation therapy, immunotherapy, targeted therapy, and hormone therapy are considered to manage the disease. Although metastatic colorectal cancer is challenging, surgery can still play a crucial role in removing isolated metastases and improving patient outcomes.

DcR3 is a soluble protein that plays a crucial role in neutralizing the biological functions of three members of the tumor necrosis factor superfamily: FasL (CD95L/TNFSF6) [5], LIGHT (CD258/TNFSF14) [6], and TNF-like molecule 1A (TL1A/VEGI/TNFSF15) [7]. Through its interactions with these ligands, DcR3 exerts regulatory effects on various cellular processes, including apoptosis, inflammation, and immune responses. A growing body of evidence has highlighted the overexpression of DcR3 in various human malignancies, including bladder urothelial carcinoma [8], breast cancer [9], pancreatic head carcinoma [10], and gastrointestinal cancer [11]. Notably, in some diseases, DcR3 has been found to exert inhibitory effects, suppressing proinflammatory cytokine and chemokine release, and activating STK10 to inhibit cell infiltration [12]. Moreover, DcR3 has been implicated in ameliorating hippocampus-dependent memory deficits and reducing amyloid plaque deposition [13]. These neuroprotective effects highlight the potential of DcR3 in maintaining brain health and mitigating neurodegenerative processes. However, despite these diverse roles observed in other diseases, limited research has been conducted on the role of DcR3 in colorectal cancer (CRC). Given its significant impact on other malignancies and its potential as a therapeutic target, it becomes crucial to elucidate the correlation between DcR3 expression and the prognosis of CRC patients.

In this study, we aimed to investigate the association between DcR3 expression and prognosis in colorectal cancer (CRC). We hypothesized that high DcR3 expression would be associated with a favorable prognosis, contrary to its well-established pro-tumorigenic functions in other cancers.

## Materials and methods

### Data acquisition and processing

In this study, we acquired comprehensive datasets and utilized various bioinformatics tools to investigate the role of DcR3 in CRC. We obtained CRC RNA sequence data containing 592 samples, along with corresponding clinical feature information, from the cBioPortal platform (http://www.cbioportal.org). cBioPortal is a renowned resource for accessing large-scale cancer genomics datasets and associated clinical data. Additionally, we downloaded CRC expression data (GSE17536) comprising 177 samples, as well as relevant clinical information, from the Gene Expression Omnibus (GEO) database (https://www.ncbi.nlm.nih.gov/geo/). To perform Gene Set Enrichment Analysis, we utilized GSEA version 4.3.2 software, which was obtained from the gsea-msigdb website (https://www.gsea-msigdb.org/gsea/index.jsp). For functional enrichment analysis, we acquired immune-related genesets from the Molecular Signatures Database (MSigDB), a well-curated collection of annotated gene sets [14, 15]. To investigate genomic alterations, we collected mutation data from TCGA through the GDC Data Portal (https://portal.gdc.cancer.gov). TCGA provides comprehensive genomic and clinical information for various cancer types. To ensure data consistency, we normalized RNA sequence counts to Transcripts per Million (TPM) values, a widely used method for quantifying and comparing gene expression levels. All data analyses were performed using R version 4.3.0, a versatile programming language commonly employed for statistical computing and graphics. R offers a plethora of packages and tools for data manipulation and analysis. For visualization and graphical representation of gene expression and correlation, we utilized Prism version 9.4.1, a widely used software tool from GraphPad.

### Immunohistochemistry (IHC)

This study included 38 patients with colorectal cancer admitted to the Third Hospital of Xi'an City between January 2017 and December 2020. There were male and female patients, with an average age of (43.3 ± 12.68) years (ranging from 18 to 62 years). The tumor TNM staging showed 16 cases in T1/T2 stage and 6 cases in T3/T4 stage, with 6 cases having lymph node metastasis. No distant organ or tissue metastasis was found in any of the cases. Cancer and adjacent tissue specimens were collected and subjected to formalin fixation and embedding for immunohistochemical testing. Pathological diagnosis was determined by two pathology experts. Pathological parameters of all cases were collected. Immunohistochemical staining was performed using the conventional streptavidin-peroxidase (SP) method. Paraffin sections of colorectal cancer tissue and adjacent tissue were routinely dewaxed and rehydrated, and antigen retrieval was performed on tissue DNA according to the first antibody requirements. 1% hydrogen peroxide was used for 20 min to eliminate the activity of endogenous peroxidase. The sections were incubated overnight at 4 °C with anti-DCR3 antibody (ab57956, working concentration 1:500) purchased from Abcam, USA. The sections were then incubated with the ready-to-use EliVision Plus secondary antibody reagent (purchased from Fuzhou Maixin Biotechnology Development Co., Ltd.) at room temperature for 25 min. Freshly prepared DAB chromogenic solution was added to the sections, and the reaction was observed under a microscope. Three fields were selected for analysis for each spot, as follows: using ImageJ software to count the total blue value a (representing cell nuclei: including dark blue and light blue), and then counting the brown positive expression b; the positive expression rate of cells was calculated as b/(a + b). The positive cell proportion was scored as 0, 1–25%, 26–50%, 51–75%, and 76–100%, corresponding to 0, 1, 2, 3, and 4 points, respectively. The staining intensity was scored as 0–3 points for negative, weak, moderate, and strong, respectively, where 0 points represented no staining, 1 point represented yellow staining, 2 points represented brown-yellow staining, and 3 points represented yellow–brown staining.The final score was obtained by multiplying the scores of the two parts, with a maximum total score of 12 points. A higher score indicates stronger positive expression.

### Analysis of immune cell abundance and tumor mutational burden (TMB)

To examine the relative abundance of DcR3 expression between normal and tumor tissues, we utilized Gene Expression Profiling Interactive Analysis (GEPIA). Additionally, to investigate the prognostic significance of DcR3, we performed survival analysis and employed a group cutoff, dividing the samples into a top 30 percent (N = 109) and low 30 percent (N = 109) based on DcR3 expression levels. To validate DcR3's prognostic impact, we utilized the Kaplan–Meier Plotter website [16] (https://kmplot.com/analysis/index.php?p=background). To delve deeper into the differences in immune cell levels between the DcR3 high and low expression groups, we assessed the immune cell abundance in all samples using CIBERSORTx [17] (https://cibersortx.stanford.edu). For this analysis, we uploaded a matrix file comprising 592 samples, with the matrix type set as Transcripts per Million (TPM). We utilized the Signature Matrix file LM22, which contains 547 genes distinguishing 22 human hematopoietic cell phenotypes [18]. Significance analysis was performed with 1000 permutations and a P value threshold of 0.05. Furthermore, we conducted an analysis of the Tumor Mutational Burden (TMB) in the DcR3 high and low expression groups in CRC using the R package maftools. This analysis aimed to explore potential differences in the number of mutations between the two groups.

### Gene set enrichment analysis (GSEA)

In order to elucidate the differences in pathway activation between the DcR3 high and DcR3 low expression groups, we conducted GSEA using two distinct methods. Firstly, we employed the GSEA software, and secondly, we utilized the R package clusterProfiler [19] for this purpose. For the GSEA analysis, we utilized gene sets from the c5.go.bp.v2023.1.Hs.entrez.gmt and c2.cp.kegg.v2023.1.Hs.entrez.gmt collections selected from the Molecular Signatures Database (MSigDB). The GSEA was performed with 1000 permutations to ensure statistical robustness and reliability. The significance threshold was set at an adjusted P value < 0.05. Through this comprehensive approach, we aimed to gain insights into the specific biological pathways that are differentially enriched in the DcR3 high and low expression groups.

### Correlation between DcR3 and clinical features

In order to ascertain the clinical relevance of DcR3, we conducted a comprehensive analysis to investigate its association with various clinical features, including age, gender, T-stage, N-stage, and M-stage. Additionally, we explored the impact of DcR3 expression on overall survival (OS) and disease-free survival (DFS) using both TCGA and GEO clinical data. To assess the survival outcomes based on DcR3 expression levels, Kaplan–Meier analysis was performed. We utilized the "survival" and "survminer" packages in R [20] to analyze and visualize the differences in OS among different DcR3 expression patterns. By undertaking these analyses, we aimed to establish the potential clinical significance of DcR3 in colorectal cancer. The correlation between DcR3 expression and various clinical features, as well as its impact on patient survival, can provide valuable insights into its role as a prognostic biomarker in CRC.

### Construction of DcR3-associated risk score model

To develop the DcR3 Associated Risk Score (DARS) model, we employed a combination of Least Absolute Shrinkage and Selection Operator (Lasso) and Cox regression analysis [21, 22]. Initially, we screened for DcR3-related genes using the TCGA-CRC RNA sequence data, considering a significance threshold of p < 0.05 and an absolute correlation value > 0.3 as the screening criteria. Next, we conducted a univariate Cox regression analysis to identify DcR3-related genes significantly associated with prognosis (p < 0.05). To further refine the gene selection and construct the DARS model, we performed Lasso-penalized Cox regression analysis using the "glmnet" R package [23]. Subsequently, a multivariate Cox regression analysis was conducted to identify the final prognostic DcR3-related genes and develop the DcR3-related gene signature. The DcR3-associated risk score was calculated using the formula: riskscore = Σ(Ci × EXPi), where EXPi represents the gene expression level of each gene and Ci represents the corresponding coefficient obtained from the multivariate Cox model [24, 25]. For the model evaluation, we used the R package "caret" to split the data into training and test sets in a 7:3 ratio. Additionally, we employed an external validation dataset, GSE17536, downloaded from GEO. To visualize the correlation between the risk score and the DARS model with clinical features, we employed a Nomogram. Furthermore, we assessed the accuracy and net benefit of the DARS model using Calibration curve and Decision Curve Analysis (DCA). Through this rigorous approach, we aimed to develop a reliable and robust DARS model that can effectively predict patient outcomes based on DcR3-associated gene expression signatures. The Nomogram, Calibration curve, and DCA analyses provide valuable insights into the clinical applicability and performance of the DARS model, facilitating its potential use as a predictive tool in colorectal cancer.

### Clinical significance of DARS

To validate the clinical significance of the DcR3-associated risk score (DARS) in the context of colorectal cancer (CRC) development, we performed GSEA. Specifically, we focused on the metabolism pathway and used the R package ggplot2 to visualize the enriched pathways. Additionally, we utilized TCGA mutation data to investigate the differences between the high-risk and low-risk groups. We presented the landscape of the top 10 genes with the highest mutation frequencies, including Missense Mutation, Intron, Silent, Frame Shift Ins, and Nonsense Mutation. Furthermore, we explored the correlation between the DARS and various clinical features to better understand its potential clinical implications in CRC. To strengthen the association between the DARS model and DcR3, we conducted the Wilcoxon test. This analysis allowed us to assess the statistical significance of the relationship between the DARS and DcR3 expression levels. Through these comprehensive analyses, we aimed to provide a deeper understanding of the clinical relevance of the DARS model in CRC. The GSEA and mutation landscape analyses shed light on potential biological pathways influenced by the DARS model, while the correlation with clinical features and the association with DcR3 help to validate its clinical significance as a potential prognostic tool in CRC.

### Data analysis and statistical analysis

Data integration and statistical analysis were conducted using R (ver. 4.3.0) and Prism 9 (ver. 9.4.1). For quantitative data, statistical significance was assessed using Student’s t-tests for normally distributed variables and the Wilcoxon rank test for non-normally distributed variables. To analyze the relationship between the DARS risk group and clinical features, we performed the Chisq-test. For datasets with three or more groups, the Kruskal–Wallis test was employed for statistical analysis. To screen for prognosis genes, we utilized Cox proportional hazards model and survival analysis. The Receiver Operating Characteristic (ROC) curve was employed to evaluate the prognostic classification performance of the risk model. An area under the curve (AUC) value over 0.5 was considered statistically significant. The overall survival data of genes were obtained from GEPIA. In all statistical analyses, a false discovery rate (FDR) or p < 0.05 was considered as the threshold for statistical significance. Through these rigorous statistical analyses, we aimed to ensure robustness and reliability in our findings. Statistical significance was appropriately assessed for different types of data, and stringent criteria were applied to identify significant results.

### Human ethics

We confirm that all methods were carried out in accordance with relevant guidelines and regulations.

We confirm that all experimental protocols were approved by a named institutional and/or licensing committee.

We confirm that informed consent was obtained from all subjects and/or their legal guardian(s).

All experimental protocols were approved by the Ethics Committee of Xi`an No.3 Hospital.

## Results

### Expression and prognosis of DcR3 in CRC

**Fig. 1:**
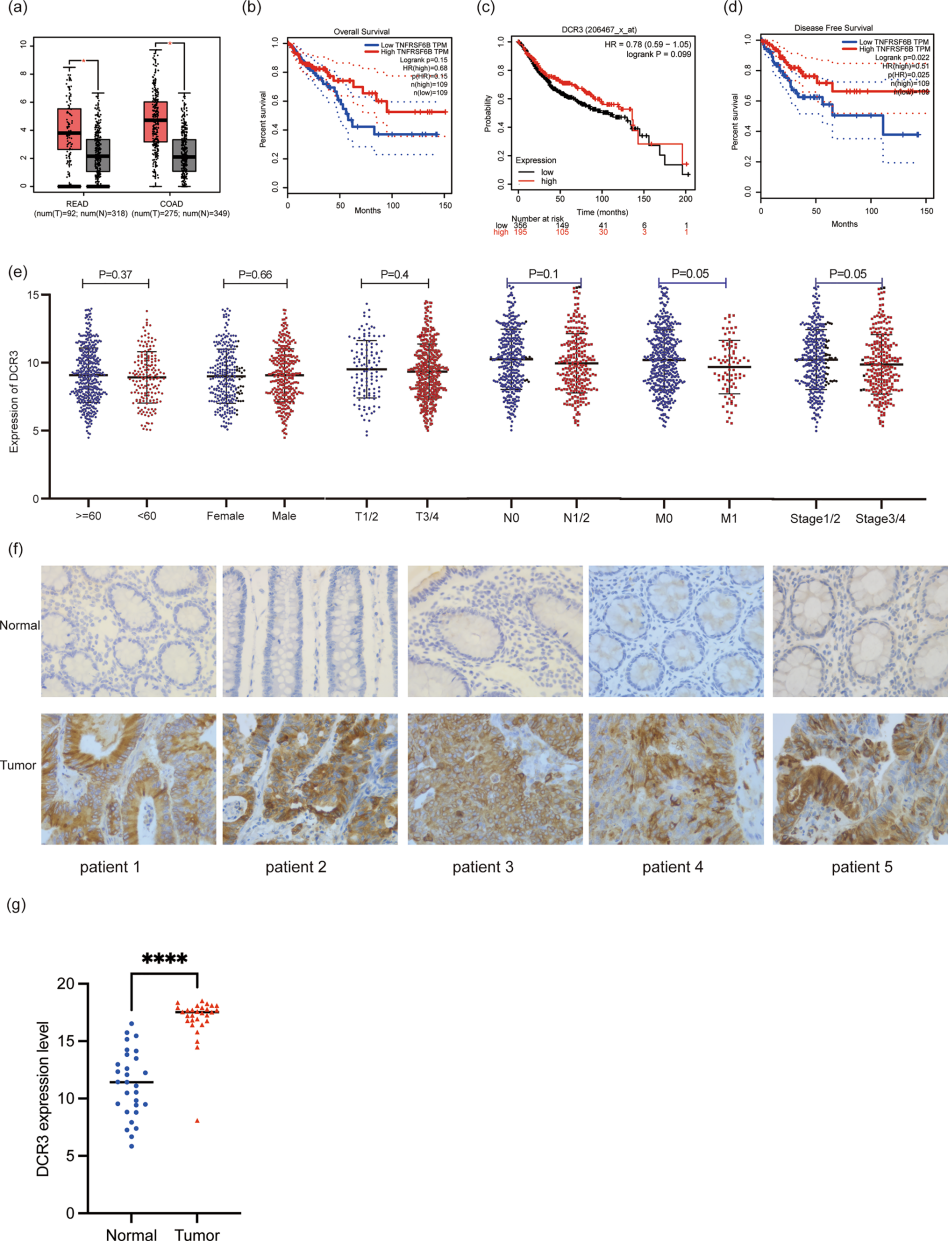
Expression and Prognosis of DcR3 in CRC. **a** DcR2 mRNA expression in READ and COAD. **b** OS of DcR3 from TCGA, KM plotter **c** and DFS of DcR3 (TCGA). **d** Expression of DcR3 in different clinical feature stage. **f** IHC straning of DcR3 in normal and tumor tissue. **e** Expression of DcR3 protein in normal and tumor tissue

DcR3 expression was significantly higher in tumor tissue compared to normal tissue (Fig. 1a). To gain further insights, we conducted survival analyses, which yielded intriguing results. High DcR3 expression was associated with a favorable prognosis in terms of both Overall Survival (OS) and Disease-Free Survival (DFS) (Fig. 1b–d). To better understand the clinical implications of DcR3, we examined its correlation with various clinical features. Interestingly, patients with high DcR3 expression were more likely to be in the M0 stage, indicating a potential association with less metastatic spread (Fig. 1e). Conversely, low DcR3 expression seemed to be linked with cancer progression to stage 3/4. Furthermore, immunohistochemical (IHC) analysis has revealed elevated levels of the DcR3 protein within tumor tissue (Figure f,g). These intriguing findings raised questions about the role of DcR3 in CRC. Despite its high expression in tumor tissue, it appears to be associated with improved survival outcomes in CRC patients. To shed light on the underlying mechanisms and potential reasons for this observation, we aim to delve deeper into the molecular and cellular factors influenced by DcR3 in CRC. Further investigation into DcR3's functional roles in CRC could provide valuable insights and contribute to our understanding of this complex disease.

### Difference in immune, TMB, and pathway of DcR3 high and low group

**Fig. 2:**
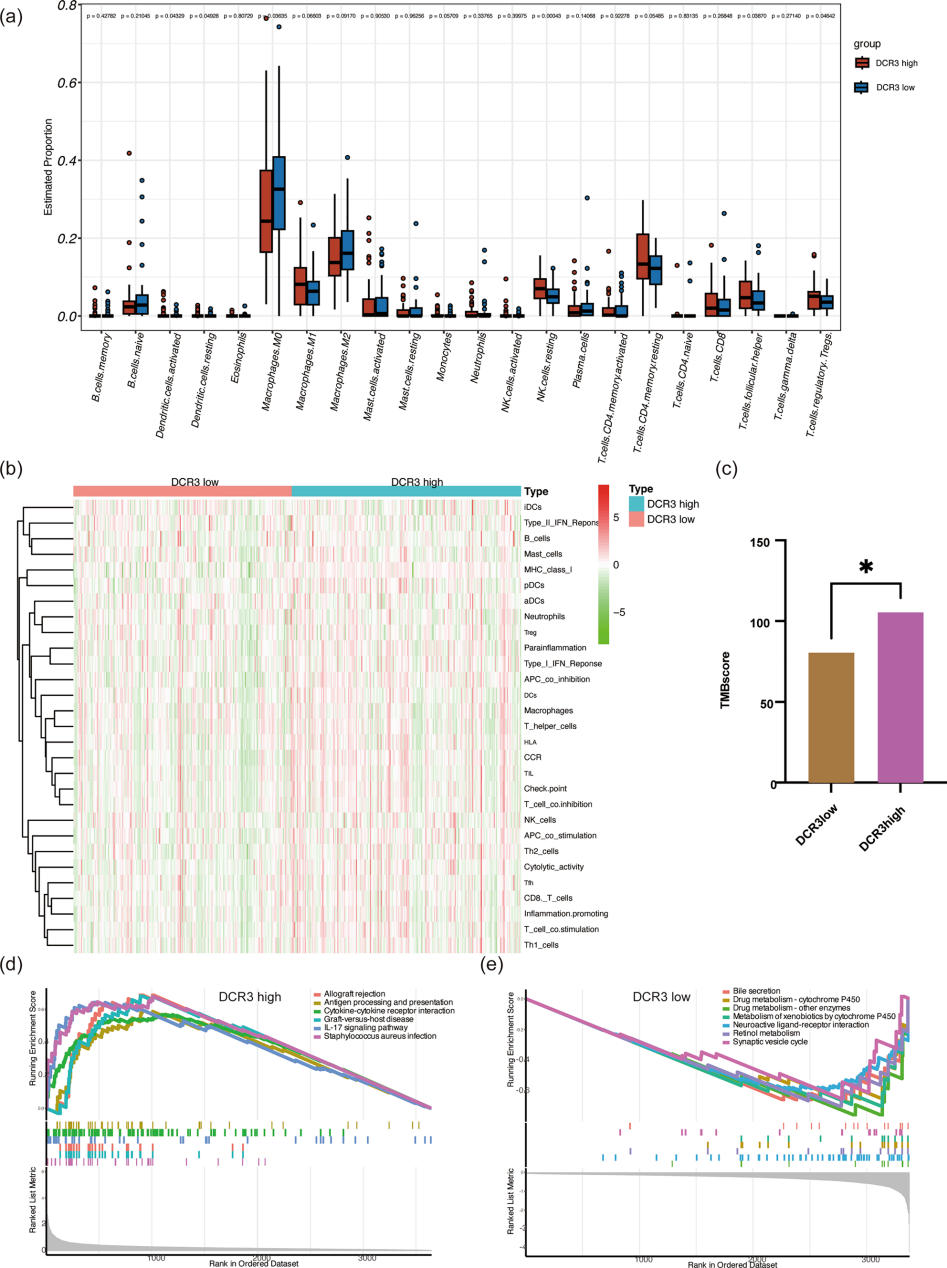
Difference in Immune, TMB, and Pathway of DcR3 High/Low Group. **a** Immune cell abundance in DcR3 High/Low Group. **b** Difference of immune feature sets in DcR3 High/Low Group. **c** TMB score in DcR3 High/Low Group. **d** Enrichment pathway in DcR3 High group and Low group (**e**)

In our efforts to understand the reasons behind the favorable prognosis observed in high DcR3 CRC patients, we examined the abundance of 22 immune cell types in the high and low DcR3 expression groups. Notably, Macrophages M0 (p = 0.03), Macrophages M2 (p = 0.09), Macrophages M1 (p = 0.06), and T cells CD4 memory resting (p = 0.05) were found to be more abundant in the high DcR3 group compared to other cells (Fig. 2a). Specifically, Macrophages M1 and T cells CD4 memory resting primarily accumulated in the DcR3 high group, while Macrophages M0 and Macrophages M2 were more prevalent in the DcR3 low group. Additionally, we evaluated the scores of 29 immune feature sets, encompassing 16 immune cells and 13 immune functions. We observed higher levels of immune-related features, such as APC_co_inhibition, APC_co_stimulation, T_cell_co_inhibition, T_cell_co_stimulation, TIL, and CD8_T_cells in the DcR3 high group (Fig. 2b). Furthermore, the DcR3 high group displayed a higher Tumor Mutational Burden (TMB) score, suggesting potential benefits for targeted cancer treatment (Fig. 2c). And GSEA revealed that pathways like Antigen processing and presentation, Cytokine-cytokine receptor interaction, and IL-17 signaling were enriched in the DcR3 high group (Fig. 2d). In contrast, the DcR3 low group exhibited enrichment in pathways such as Bile secretion, Drug metabolism-cytochrome P450, Drug metabolism-other enzymes, and Metabolism of xenobiotics by cytochrome P450 (Fig. 2e). These findings suggest that the immune system may play a crucial role in the favorable prognosis observed in the DcR3 high group, while metabolism pathways may be disturbed in the DcR3 low group. These results offer valuable insights into the potential mechanisms underlying the favorable outcomes observed in high DcR3 CRC patients and provide a basis for further exploration of immune-related pathways and therapeutic opportunities in colorectal cancer management.

### DcR3-associated risk score (DARS) model

**Fig. 3:**
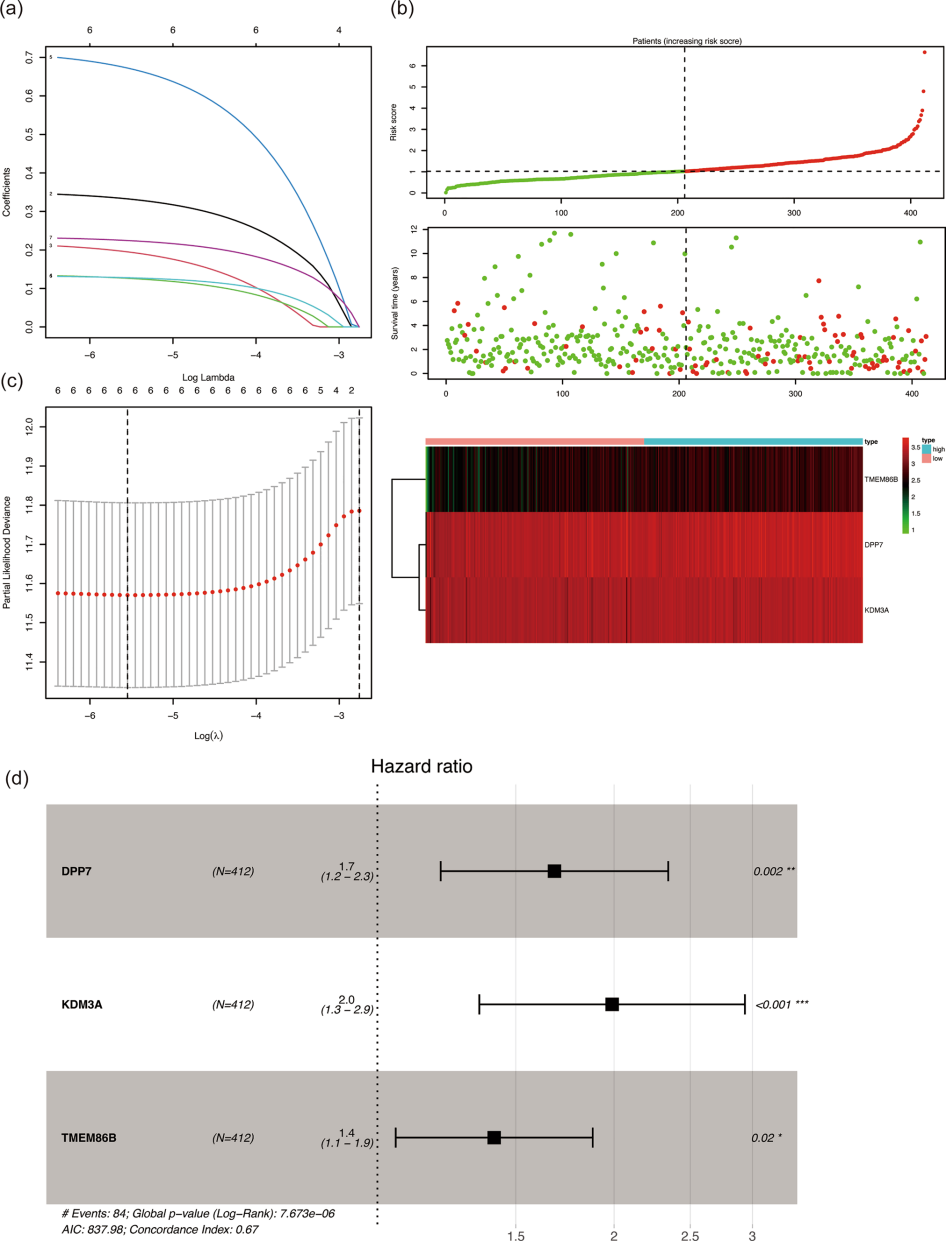
DcR3-Associated Risk Score (DARS) Model. **a** Lasso coefficient profiles of the 7 DcR3-Associated genes. **b** Survival time and status of High/Low Risk patients and expression of three genes in High/Low Risk group. **c** LASSO regression analysis was used to prevent the overfitting effects of the model. **d** The screened genes were brought into multivariate Cox regression analysis and constructed the DARS Model

To delve deeper into the potential functions of DcR3, we sought to construct a riskscore model that would be associated with DcR3. In the initial step, we employed the Pearson correlation method to identify 769 genes that were correlated with DcR3 (r > 0.3 or r < 0.3, p < 0.05). Next, a univariate Cox regression analysis was performed on the training set, leading us to select 7 genes (ABCA7, DPP7, HDAC10, HMHA1, KDM3A, NFKB2, and TMEM86B) for further analysis through the Lasso method (Fig. 3a, c). In the training cohort, we calculated the riskscore for each sample and categorized them into either high or low-risk groups based on the median riskscore. The results demonstrated that the high-risk group had a higher mortality rate, whereas the low-risk group had a higher survival rate (Fig. 3b). We further validated these findings in the test group, where we observed a similar pattern with the high-risk group exhibiting increased mortality (Figure S1). Subsequently, utilizing Lasso and Multivariate Cox regression analysis, we successfully identified a refined riskscore model comprising 3 genes (DPP7, KDM3A, and TMEM86B) (Fig. 3d). This model, named DARS, allowed us to calculate the riskscore using the formula: riskscore = (0.52 × expression of DPP7) + (0.69 × expression of KDM3A) + (0.34 × expression of TMEM86B). The DARS model provides a promising tool to assess risk levels and predict patient outcomes based on the expression of these 3 genes. By incorporating the DARS model, we aim to gain better insights into the potential impact of DcR3 on CRC prognosis, as well as identify potential therapeutic targets for personalized cancer management.

### Estimation of risk model and establishment of nomograms

**Fig. 4:**
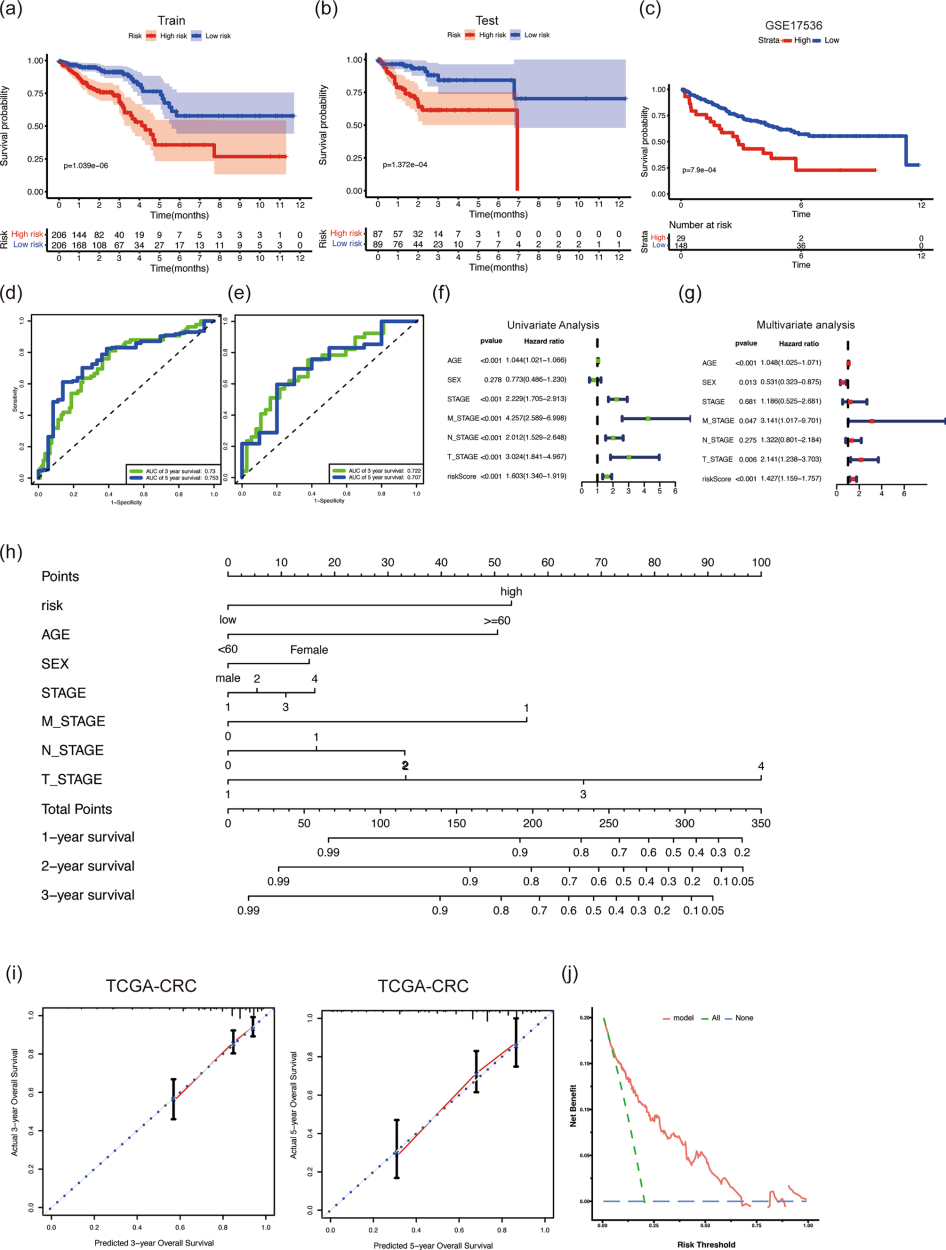
Estimation of Risk Model and Establishment of Nomograms. **a** Prognosis of DARS in Train set, Test set (**b**) and GEO set (**c**). **d**, **e** ROC curve about 3-year and 5-year survival. **f**, **g** Univariate and Multivariate Cox regression analyses. **h** Nomograms including riskScore, age, sex, stage, M_STAGE, N_STAGE, and T_STAGE. **i** Calibration curve of DARS in 3-year and 5-year. **j** Evaluation of the clinical usefulness of the DARS

To thoroughly evaluate the performance of the riskscore model, we conducted survival analyses and ROC curve analysis. Our results consistently showed that the high-risk group exhibited poorer prognosis in both the train and test sets (Fig. 4a, b). External validation with the GSE17536 dataset also confirmed the association between the high-risk group and unfavorable prognosis (Fig. 4c). ROC curve analysis further demonstrated the accuracy of the riskscore model, with AUC values of 0.73 and 0.753 for 3-year and 5-year survival in the train set, respectively. The AUC values for the 3-year and 5-year survival in the test set were 0.722 and 0.707, respectively (Fig. 4d, e). Next, we performed univariate and multivariate Cox regression analyses using TCGA and GSE17536 clinical data to explore the prognostic significance of the risk model alongside other clinical factors. Univariate Cox regression revealed that age, stage, M_STAGE, N_STAGE, T_STAGE, and riskScore were significantly associated with prognosis in TCGA data, while stage, grade, and riskScore were associated with prognosis in GSE17536 data (Fig. 4f and Figure S2a). Multivariate Cox regression analysis in TCGA data identified age, sex, M_STAGE, T_STAGE, and riskScore as independent prognostic factors, while in GSE17536 data, stage and riskScore were the independent prognostic factors (Fig. 4g and Figure S2a). To provide clinicians with a practical tool for predicting survival risk, we developed nomograms for both TCGA and GEO cohorts. These nomograms integrated riskScore, age, sex, stage, M_STAGE, N_STAGE, and T_STAGE for the TCGA cohort and riskScore, age, gender, stage, and grade for the GEO cohort (Fig. 4h and Figure S2b). Furthermore, we validated the accuracy and net benefit of the DARS model using Calibration curve and Decision Curve Analysis (DCA) in both TCGA and GEO cohorts (Fig. 4i, j and Figure S2c). The results confirmed the stability and clinical utility of our riskscore model. It can serve as an independent prognostic indicator, aiding clinicians in more accurately predicting patient outcomes and potentially guiding treatment decisions for CRC patients.

### Assessment of correlation between DARS model and clinical features

**Fig. 5:**
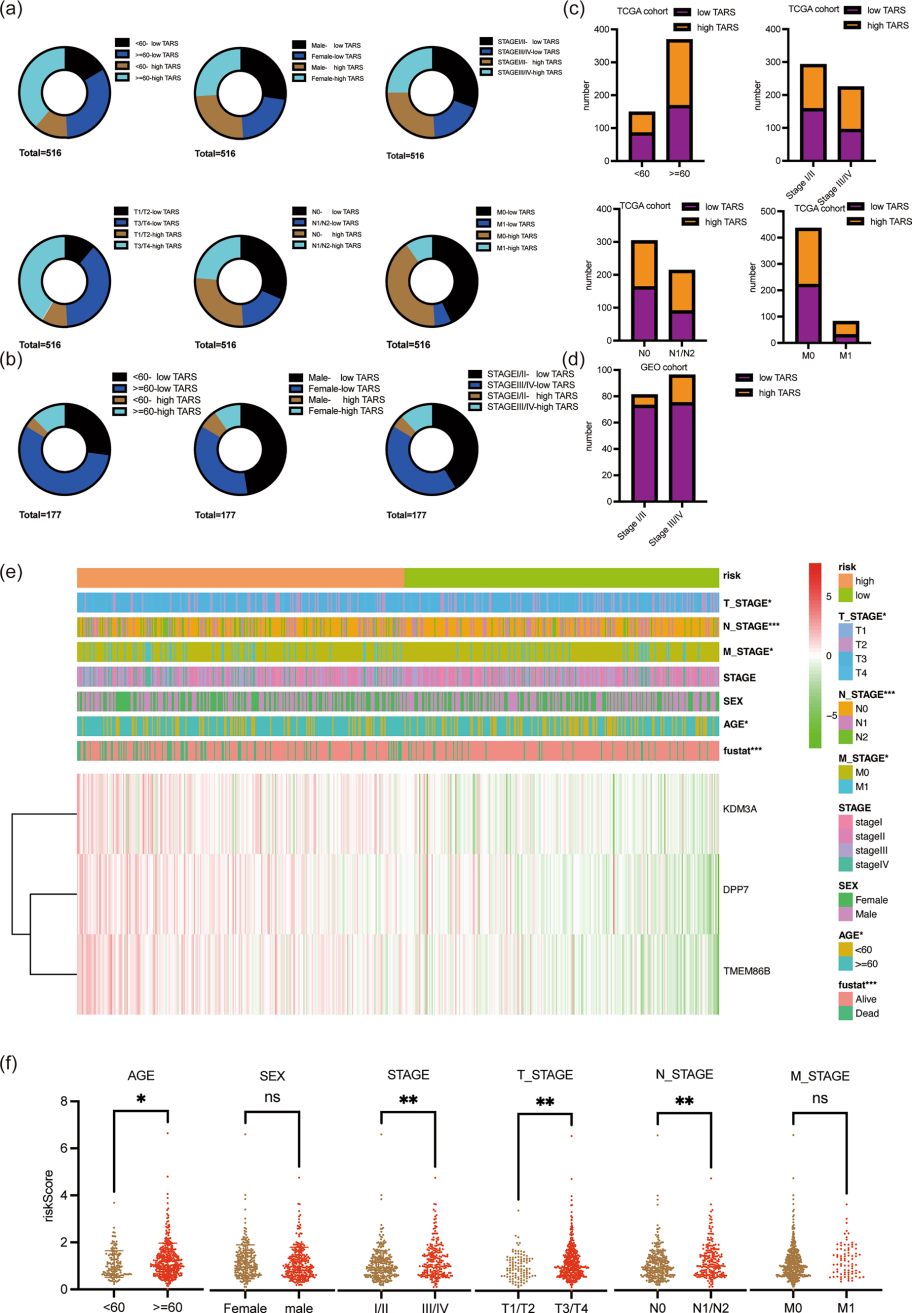
Assessment of Correlation between DARS Model and Clinical Features. Proportion of high and low risk groups was profiled for each clinical feature in TCGA cohort (**a**, **c**) and GEO cohort (**b**, **d**). **e**, **f** Correlation between DARS and clinical features

Clinical features serve as important indicators for the development of cancers [26]. To gain deeper insights into the association between the DARS model and CRC, we conducted a Chisq-test to analyze the relationship between the DARS risk group and various clinical features in CRC. The distribution of high and low risk groups was profiled for each clinical feature (Fig. 5a, b). Our findings indicated that the high DARS group was positively correlated with age, suggesting that higher DARS may be associated with older age in CRC patients. Additionally, high DARS was found to promote cancer development from stage I/II to stage III/IV, implying that elevated DARS might be linked to disease progression (Fig. 5c). Moreover, the DARS was associated with M_STAGE and N_STAGE in the TCGA cohort, indicating that high DARS could be indicative of tumor metastasis and increased lymph node involvement (Fig. 5c). These results were further validated in the GEO cohort, providing additional support for the impact of DARS on CRC development (Fig. 5d). More detailed statistics on the correlation between DARS and clinical features can be found in Table 1. This table presents comprehensive data on the relationship between clinical features and the DARS group across different cohorts, including train, test, TCGA, and GSE17536. Furthermore, we analyzed the difference in riskScore across different stages of clinical features. The results aligned with our previous findings, demonstrating that patients over 60 years of age and those with clinical stages at III/IV had higher risks (Fig. 5e, f). These findings are in line with well-established clinical facts, further strengthening the significance of our DARS model as a reliable prognostic tool for CRC patients. In conclusion, the correlation analysis between the DARS model and clinical features sheds light on the potential impact of DARS on CRC development and progression. By identifying these associations, our model proves to be a valuable asset for predicting patient outcomes and guiding treatment decisions in CRC management.

**Table 1:** Correlation between DARS and clinical features

		Train			Test			TCGA CRC			GSE17536		
		Low TARS	High TARS	pvalue	Low TARS	High TARS	pvalue	Low TARS	High TARS	pvalue	Low TARS	High TARS	pvalue
AGE	< 60	55	49		30	14		85	63		48	7	
	≥ 60	119	141	0.22	49	59	0.011	168	200	0.015	100	22	0.377
SEX	Male	91	99		50	30		141	129		84	12	
	Female	83	91	0.971	29	43	0.006	112	134	0.129	64	17	0.129
STAGE	I/II	117	100		41	34		158	134		73	8	
	III/IV	57	90	0.005	38	39	0.512	95	129	0.008	75	21	0.032
M_STAGE	M0	156	157		66	56		222	213				
	M1	18	33	0.054	13	17	0.29	31	50	0.035			
N_STAGE	N0	121	105		42	35		163	140				
	N1/N2	53	85	0.005	37	38	0.52	90	123	0.01			
T_STAGE	T1/T2	36	38		22	11		58	49				
	T3/T4	138	152	0.87	57	62	0.056	195	214	0.229			

### Mutation and enrichment analysis between high and low riskscore groups

**Fig. 6:**
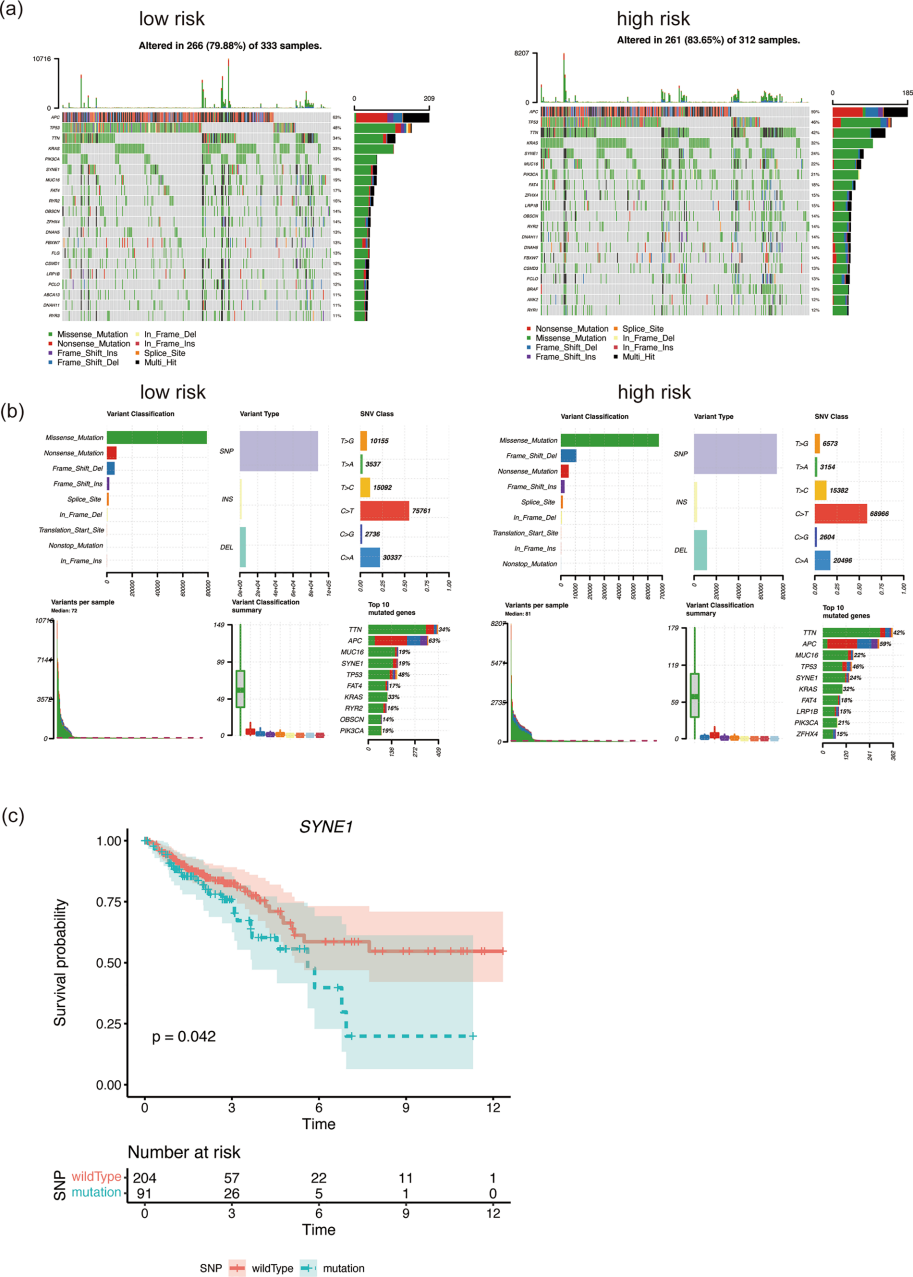
Mutation and Enrichment Analysis between High and Low Riskscore Groups. Landscape of mutation in High/Low risk groups (**a**, **b**). **c** Survival analysis of SYNE1

**Fig. 7:**
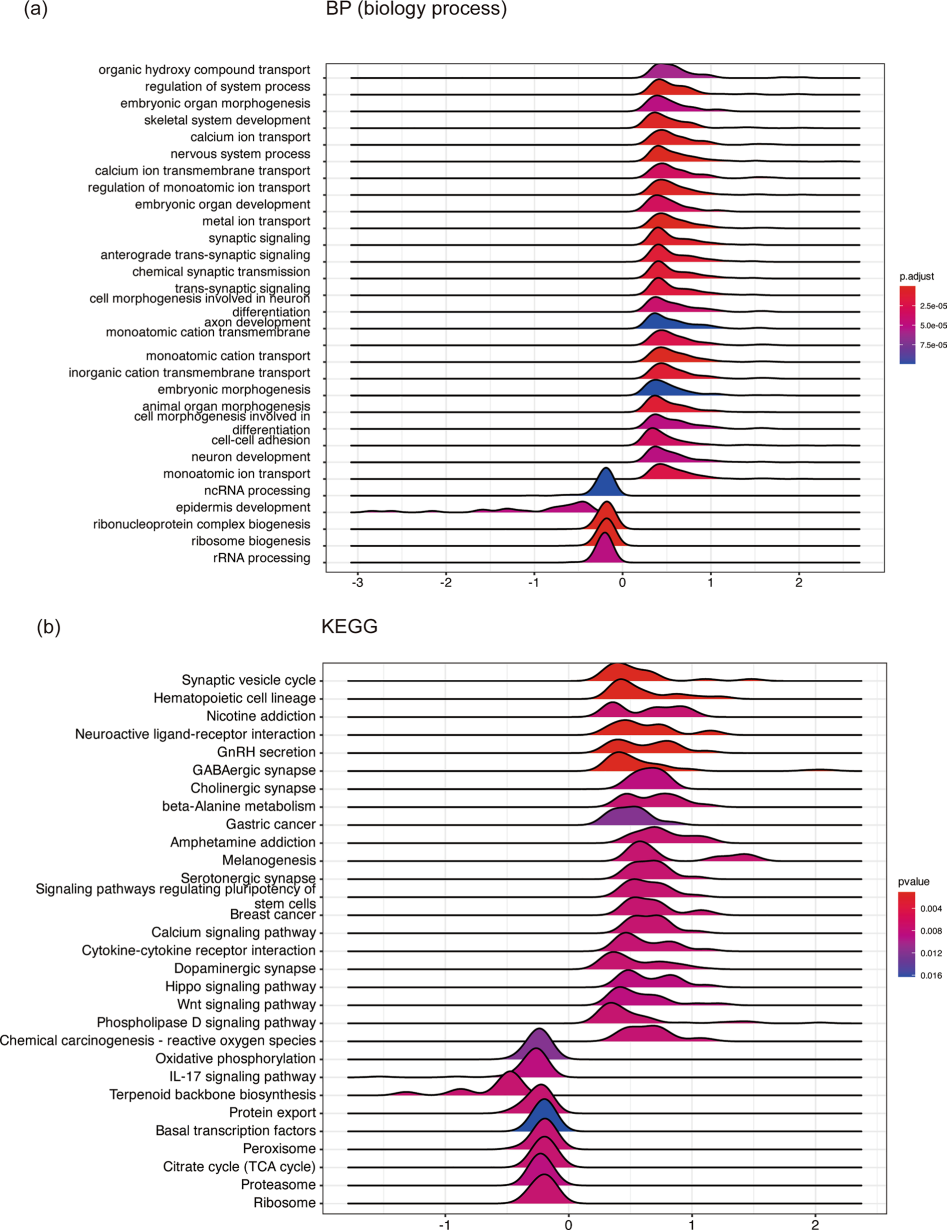
Enrichments of pathway in High/Low risk groups of DARS. **a** Biology process. **b** KEGG

To explore the differences in mutation patterns, we obtained CRC mutation data from TCGA. We identified the top 20 genes with the highest mutation frequency in both the high and low riskscore groups (Fig. 6a). The mutation types included Nonsense Mutation, Missense Mutation, Frame Shift Del, Frame Shift Ins, Splice Site, In Frame Del, In Frame Ins, and Multi Hit. Nonsense Mutation emerged as the predominant cause of gene mutation. In the low riskscore group, the top 10 mutated genes were TTN (34%), APC (63%), MUC16 (19%), SYNE1 (19%), TP53 (48%), FAT4 (17%), KRAS (33%), RYR2 (16%), OBSCN (14%), and PIK3CA (19%). Conversely, in the high riskscore group, the top 10 mutated genes were TTN (42%), APC (59%), MUC16 (22%), SYNE1 (24%), TP53 (46%), FAT4 (18%), KRAS (32%), LRP1B (15%), ZFHX4 (15%), and PIK3CA (21%) (Fig. 6b). We conducted survival analysis to investigate the correlation between mutations and prognosis, revealing that mutations in SYNE1 were associated with poor prognosis (Fig. 6c). These results indicated that the high riskscore group had a higher mutation frequency, and specifically, mutations in SYNE1 were linked to decreased survival time. Additionally, we performed enrichment analysis to uncover the different pathways between the high and low riskscore groups. We focused on both biological processes (BP) and the Kyoto Encyclopedia of Genes and Genomes (KEGG). The pathways enriched in the high riskscore group included organic hydroxy compound transport, regulation of system process, embryonic organ morphogenesis, skeletal system development, and calcium ion transport, among others (Fig. 7a). In contrast, the pathways enriched in the low riskscore group involved ncRNA processing, epidermis development, ribonucleoprotein complex biogenesis, ribosome biogenesis, and rRNA processing (Fig. 7a). KEGG analysis indicated that the high riskscore group showed enrichment in pathways such as the Synaptic vesicle cycle, Hematopoietic cell lineage, Nicotine addiction, GnRH secretion, and Neuroactive ligand-receptor interaction, while the low riskscore group displayed enrichment in pathways like Oxidative phosphorylation, Chemical carcinogenesis—reactive oxygen species, Protein export, IL-17 signaling pathway, and Basal transcription factors (Fig. 7b). Notably, the low riskscore group exhibited activation of the IL-17 signaling pathway. This suggests that the low riskscore group may promote immune reactions through the IL-17 signaling pathway, potentially contributing to their more favorable prognosis. The combination of mutation and enrichment analysis provides valuable insights into the underlying biological mechanisms and potential therapeutic targets associated with the DARS model in CRC.

### Correlation between DcR3 and riskScore group

**Fig. 8:**
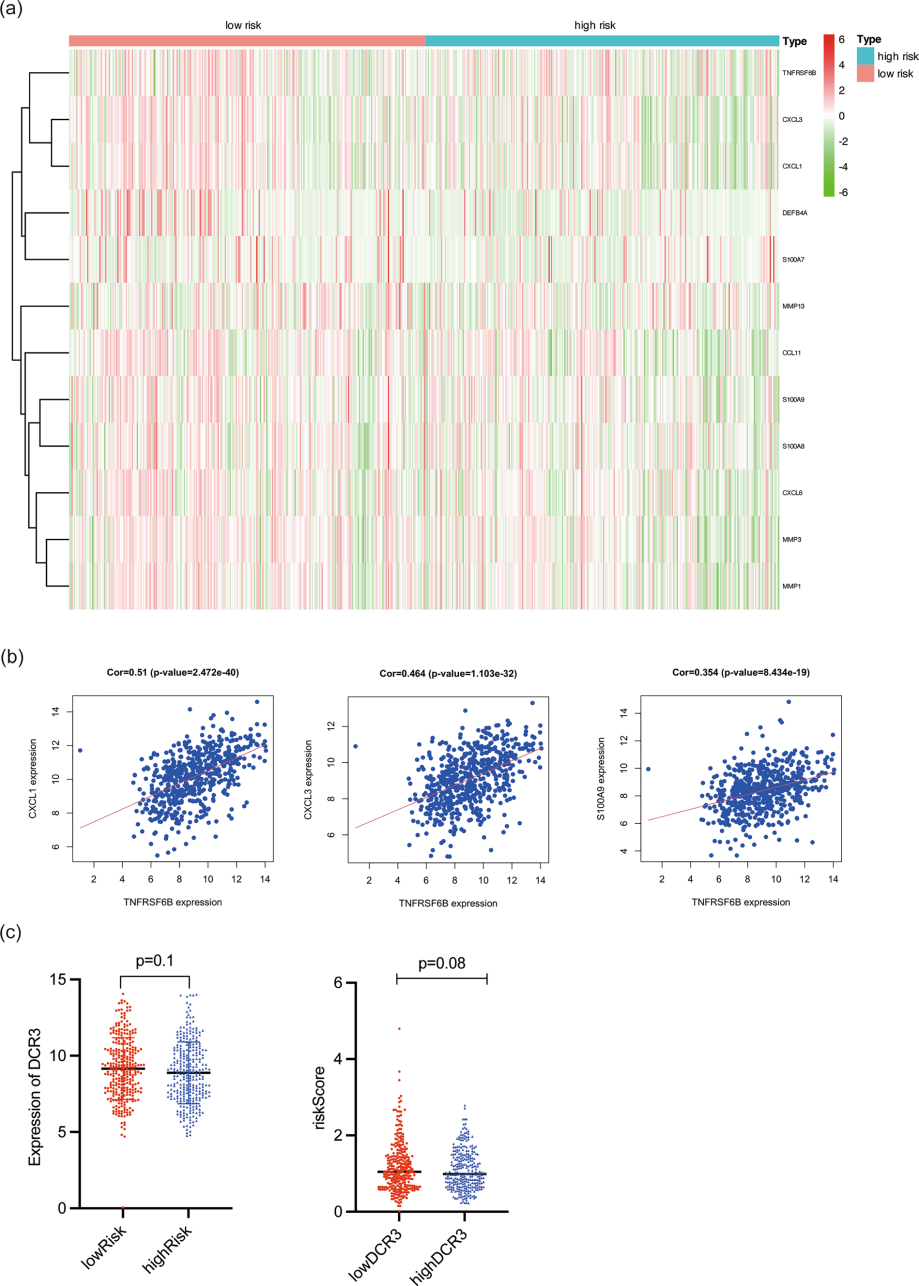
Correlation between DcR3 and riskScore group of DARS. **a** Heatmap of DcR3 and genes in IL-17 signaling pathway. **b** Correlation between DcR3 and genes. **c** Correlation between DcR3 and riskScore

The observed differences in survival outcomes between high DcR3 and low DcR3 patients, along with the correlation analysis with the IL-17 signaling pathway, provide valuable insights into the potential reasons behind the contrasting prognosis in CRC patients. Firstly, the analysis of IL-17 related genes revealed that MMP13, CXCL3, MMP3, CCL11, CXCL1, CXCL6, MMP1, S100A9, S100A8, DEFB4A, S100A7, and TNFRSF6B exhibited higher expression levels in the low riskScore group (Fig. 8a). Subsequently, the correlation analysis demonstrated that DcR3 expression was positively correlated with the expression of CXCL3 (r = 0.464, p < 0.001), CXCL1 (r = 0.51, p < 0.001), and S100A9 (r = 0.354, p < 0.001) (Fig. 8b). This suggests that DcR3 may play a role in activating the immune system by modulating the expression of these IL-17 related genes. The upregulation of these genes in the low riskScore group might contribute to the better prognosis observed in these patients. Additionally, the analysis of DcR3 expression in the high riskScore group showed that DcR3 levels were relatively low (p = 0.1), and conversely, in the high DcR3 expression group, the riskScore was also relatively low (p = 0.08) (Fig. 8c). This further supports the notion that high DcR3 expression is associated with a more favorable prognosis, as evidenced by the lower riskScore in these patients. Collectively, these findings suggest that the interaction between DcR3 and the IL-17 signaling pathway may be a key factor in determining the survival outcomes of CRC patients. DcR3 might act as an activator of the immune system through its regulation of IL-17 related genes, leading to improved prognosis in CRC patients with higher DcR3 expression. Understanding the interplay between DcR3 and the IL-17 signaling pathway can provide important insights into the molecular mechanisms underlying CRC development and progression, and it highlights DcR3 as a potential target for further investigation and therapeutic interventions.

## Discussion

TNFRSF6B (TNF receptor superfamily member 6b), also known as DcR3, is a member of the tumor necrosis factor receptor superfamily [5, 27]. Numerous studies have consistently reported that DcR3 is highly expressed in tumor tissue compared to normal tissue, suggesting its role as a positive regulator in cancer development. Through various mechanisms, including inhibiting FasL- and LIGHT-mediated cell death and promoting tumor cell inflammation and migration, DcR3 has been implicated in cancer progression across different types of malignancies [28–34]. However, recent insights have revealed a paradoxical association between high DcR3 expression and favorable prognosis in colorectal cancer (CRC), challenging our conventional understanding.

In our study, we observed elevated DcR3 expression in tumor tissue compared to adjacent normal tissue in CRC. Contrary to expectations, high DcR3 expression correlated with improved survival outcomes, lower rates of metastatic disease, and lower cancer stage, suggesting a potential inhibitory effect on tumor development. To elucidate the underlying mechanisms, we categorized CRC patients based on DcR3 expression levels and conducted comprehensive analyses. Our findings indicated a potential role of DcR3 in activating the immune system, as evidenced by the enrichment of immune-related pathways, particularly the IL-17 signaling pathway, in patients with high DcR3 expression. Further analysis using riskScore modeling revealed that high DcR3 expression was associated with a low risk of adverse outcomes, corroborating its potential as a prognostic marker in CRC.

The phenomenon of certain molecules, including DcR3, exhibiting differential prognostic implications in cancer underscores the complexity of tumor biology and treatment response. Similar observations have been reported for other genes such as CXCL11, Plk1, and CD177, where high expression levels are unexpectedly associated with better clinical outcomes in specific cancer types [35–37]. These paradoxical effects challenge traditional paradigms and highlight the need for a nuanced understanding of gene expression patterns and their functional implications in cancer biology.

Our study contributes novel insights into the role of DcR3 in CRC and its potential as a prognostic marker and therapeutic target. By unraveling the intricate interplay between DcR3 expression, immune activation, and tumor progression, we provide a basis for further research aimed at leveraging DcR3 modulation for improved patient outcomes. However, our study has limitations, including sample size constraints and reliance on specific datasets, necessitating validation in larger cohorts and diverse clinical settings. Nevertheless, our findings pave the way for personalized therapeutic strategies tailored to individual gene expression profiles and tumor microenvironments, ultimately optimizing treatment efficacy for CRC patients.

### Supplementary Information

Supplementary material 1. Figure S1. DcR3-Associated Risk Score (DARS) Model Test set. (a) Survival time and status of High/Low Risk patients and expression of three genes in High/Low Risk group in Test set. (b) Univariate and Multivariate Cox regression analyses in Test set.

Supplementary material 2. Figure S2. Estimation of Risk Model and Establishment of Nomograms in GEO set. (a) Univariate and Multivariate Cox regression analyses in Test set. (b) Nomograms including risk, age, gender, stage and grade in GEO set. (c) Calibration curve of DARS in 3-year and 5-year and Evaluation of the clinical usefulness of the DARS in GEO set.

## Data Availability

The datasets analyzed during the current study are publicly available in the cBioPortal platform (http://www.cbioportal.org) and GEO (Gene Expression Omnibus) repositories. The cBioPortal platform data can be accessed at http://www.cbioportal.org, and GEO data can be accessed at https://www.ncbi.nlm.nih.gov/geo/. The specific datasets used include TCGA-COAD and GEO series GSE17536.
