# Supplementary figures and images for: DcR3-associated risk score: correlating better prognosis and enhanced predictive power in colorectal cancer
Source: Discov Oncol. 2024 Jun 19;15:233. doi: 10.1007/s12672-024-01082-1 (PMC11189376; doi:10.1007/s12672-024-01082-1)

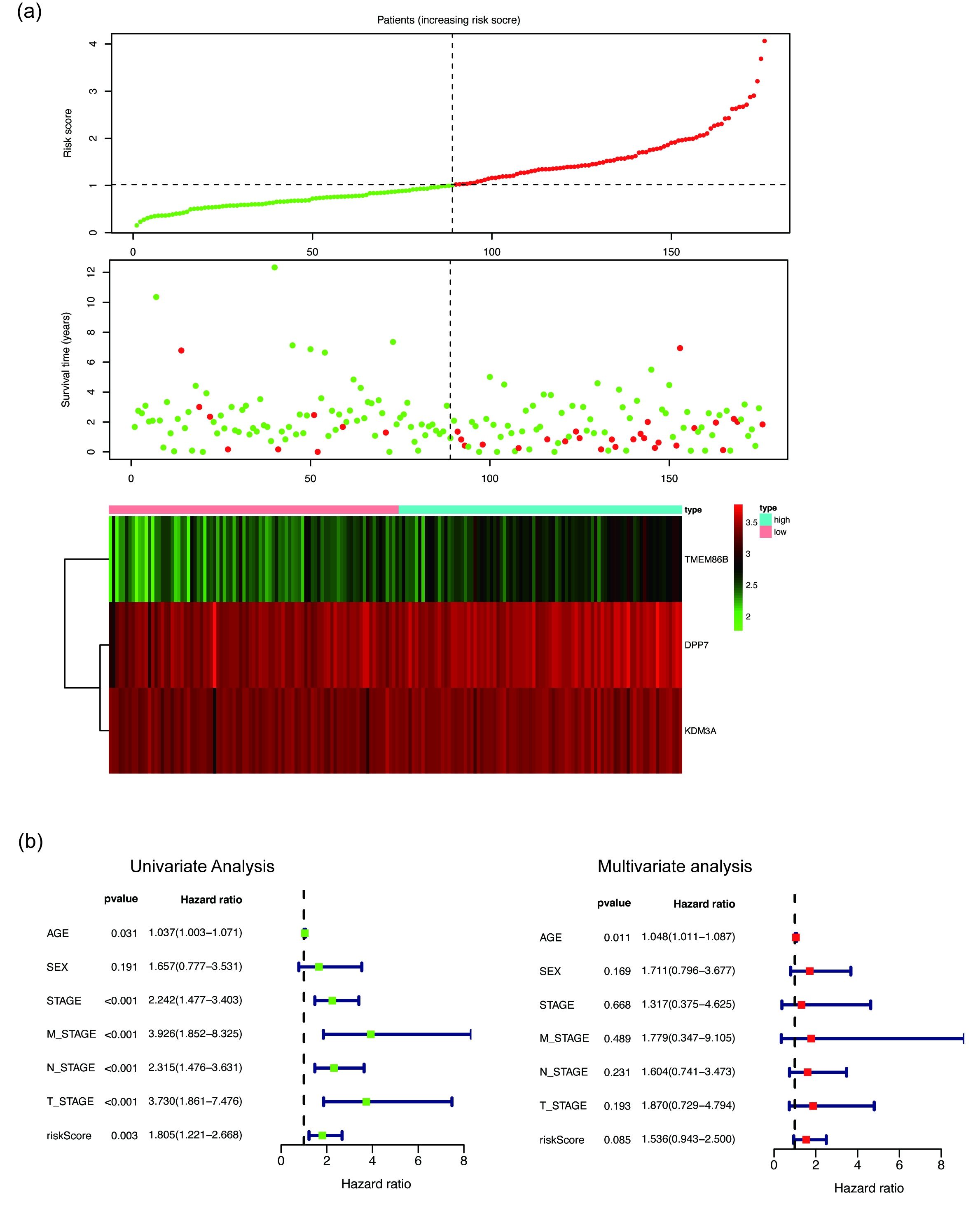

Supplement: Supplementary file 1 — Supplementary material 1. Figure S1. DcR3-Associated Risk Score (DARS) Model Test set. (a) Survival time and status of High/Low Risk patients and expression of three genes in High/Low Risk group in Test set. (b) Univariate and Multivariate Cox regression analyses in Test set. [file 12672_2024_1082_MOESM1_ESM.tif]

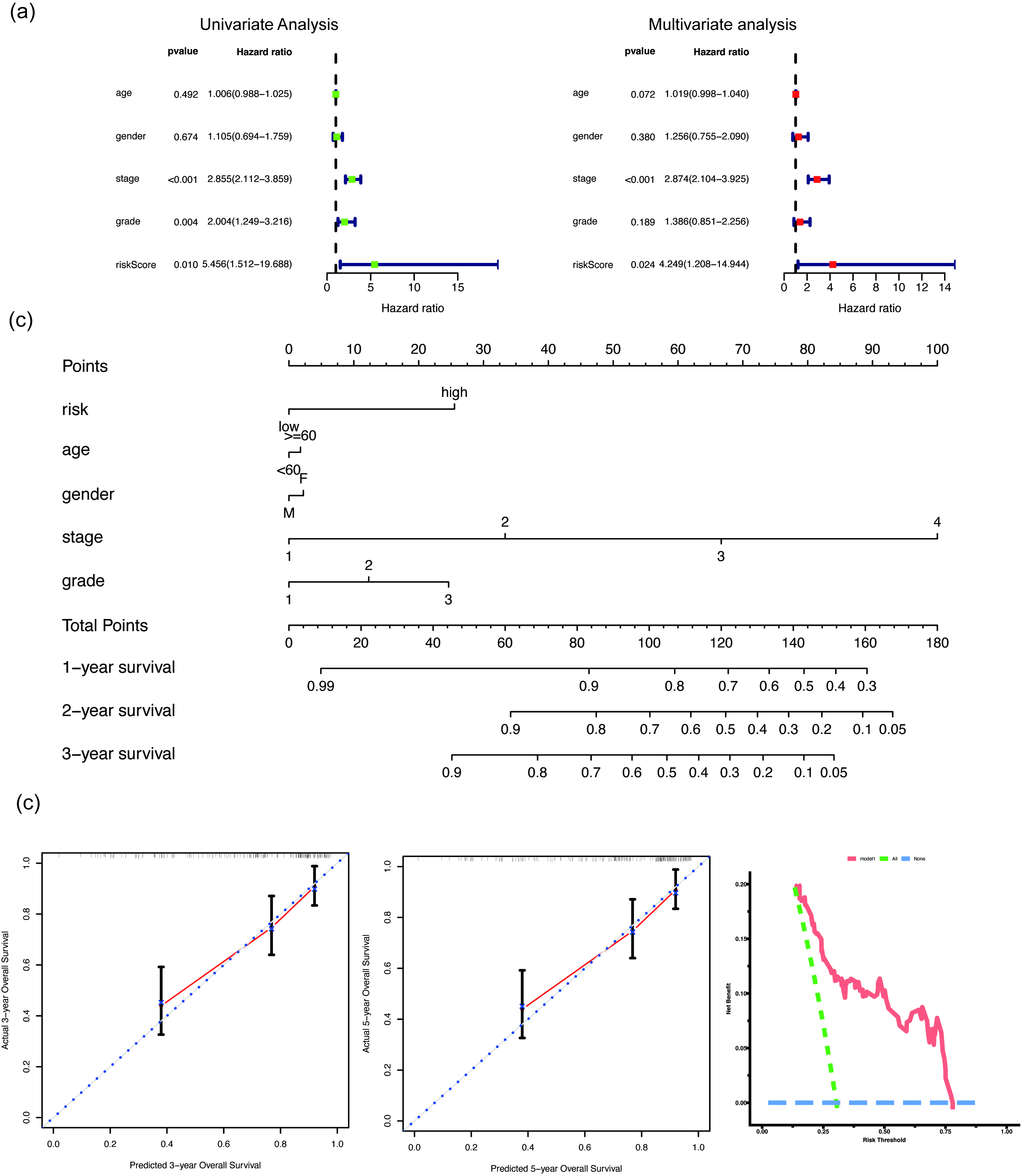

Supplement: Supplementary file 2 — Supplementary material 2. Figure S2. Estimation of Risk Model and Establishment of Nomograms in GEO set. (a) Univariate and Multivariate Cox regression analyses in Test set. (b) Nomograms including risk, age, gender, stage and grade in GEO set. (c) Calibration curve of DARS in 3-year and 5-year and Evaluation of the clinical usefulness of the DARS in GEO set. [file 12672_2024_1082_MOESM2_ESM.tif]
